# Supplementary material for: Reference compounds for characterizing cellular injury in high-content cellular morphology assays
Source: Nat Commun. 2023 Mar 13;14:1364. doi: 10.1038/s41467-023-36829-x (PMC10011410; doi:10.1038/s41467-023-36829-x)
Supplement: Supplementary file 1 — Supplementary Information [file 41467_2023_36829_MOESM1_ESM.pdf]

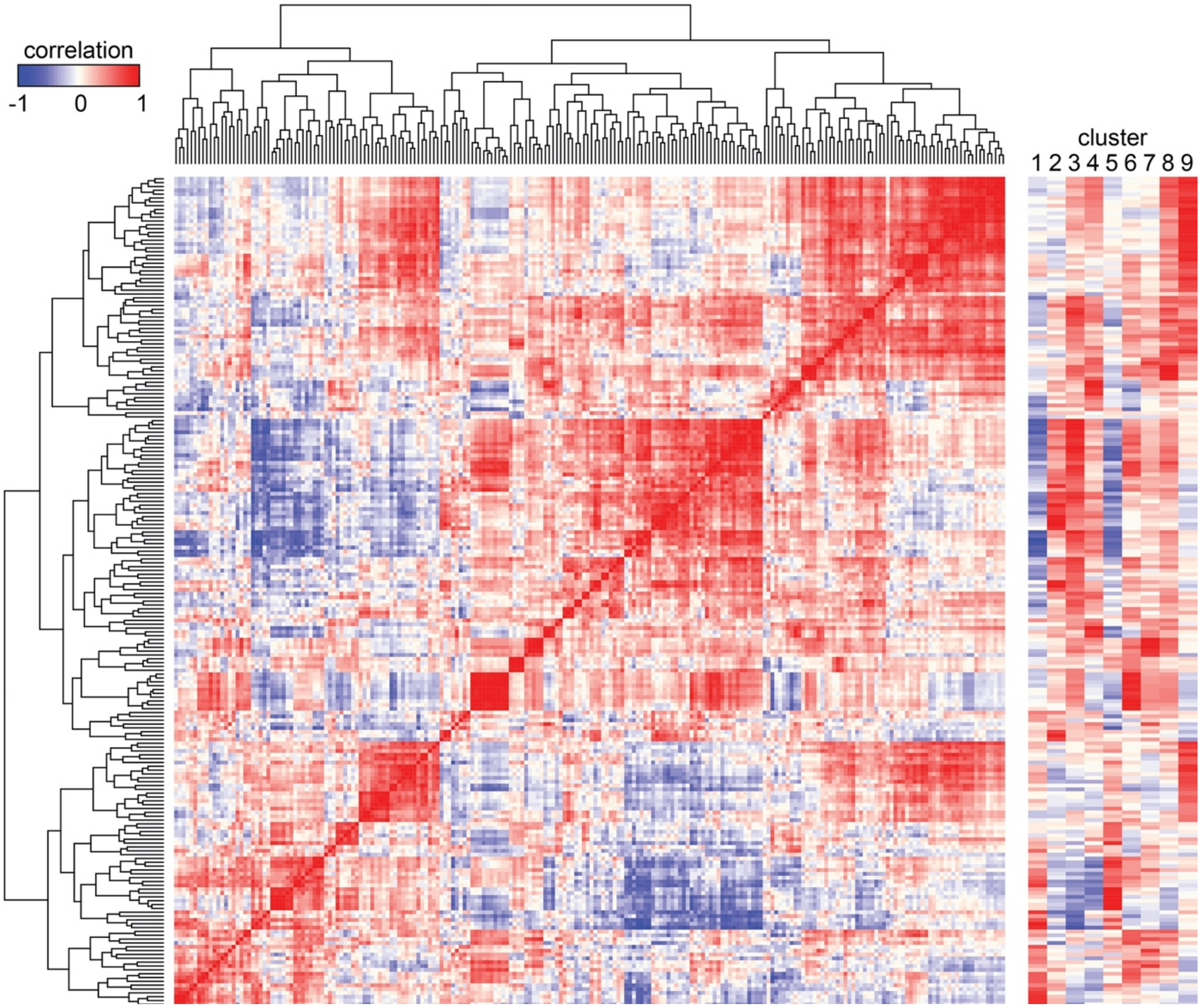

Supplementary Figure 1. Summary of cell painting compound clustering. Hierarchical clustering was performed on compounds (**1-171**, **455-501**) using as their distance 1 - *x*, where *x* is the pairwise Pearson correlation coefficient between compound treatments. The square heatmap depicts these pairwise correlation coefficients and is sorted according to the hierarchical clustering. The accompanying non-clustered heatmap to the right depicts the Pearson correlation coefficient of these same compounds against the consensus profiles for the 9 clusters. Note that the correlation coefficients were calculated using the average profile for each compound across all treatment concentrations. Data are from 24 h compound treatment times. Source data are provided as a Source Data file.

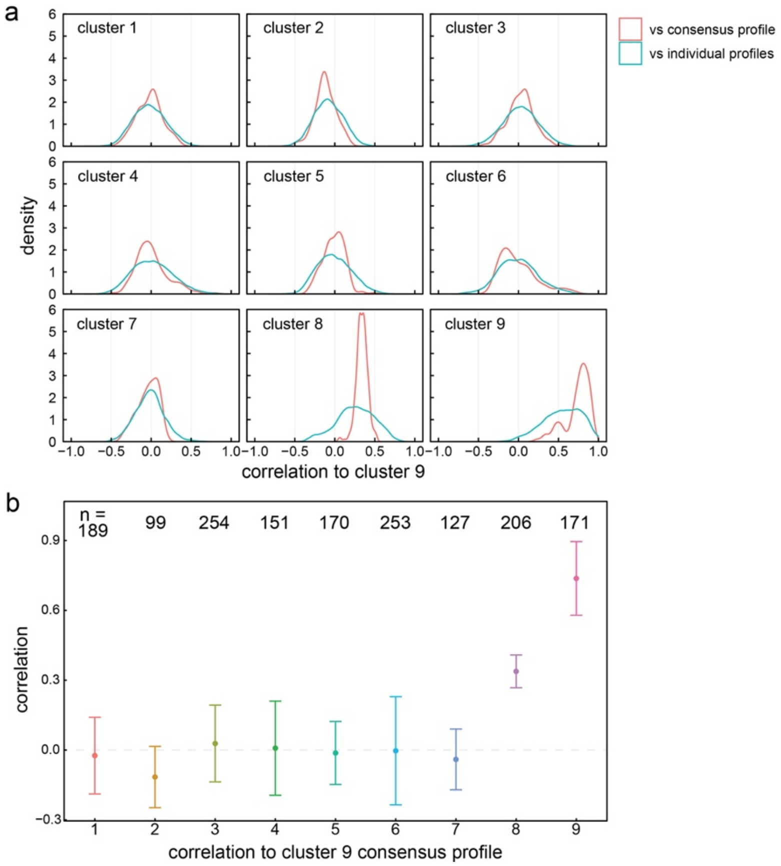

Supplementary Figure 2. Calculation of cellular injury phenotype. (a) The cell injury phenotype is calculated as the average signature of cluster 9, where the average value for each feature across all cluster 9 members is taken, and the vector comprising these average values is used as the cell injury phenotype. This average profile can be considered the central point of the cloud of cell injury phenotypes in multidimensional space. While the profiles of compounds belonging to cluster 9 were somewhat diverse, their correlation to this consensus cell injury phenotype was more robust compared to the pairwise correlation to all compounds belonging to cluster. There is a significant improvement in the consistency of these correlations when comparing the consensus cell injury phenotype to individual compounds belonging to cluster 8. (b) Comparing the correlations of all compounds to this consensus cell injury phenotype, we find that those belonging to cluster 9 have the highest correlations to the consensus profile, suggesting that this metric can be used to predict MLI compounds that might also induce similar cell injury phenotypes. Data are mean ± SD (precise *n* values denoted in figure). Source data are provided as a Source Data file.

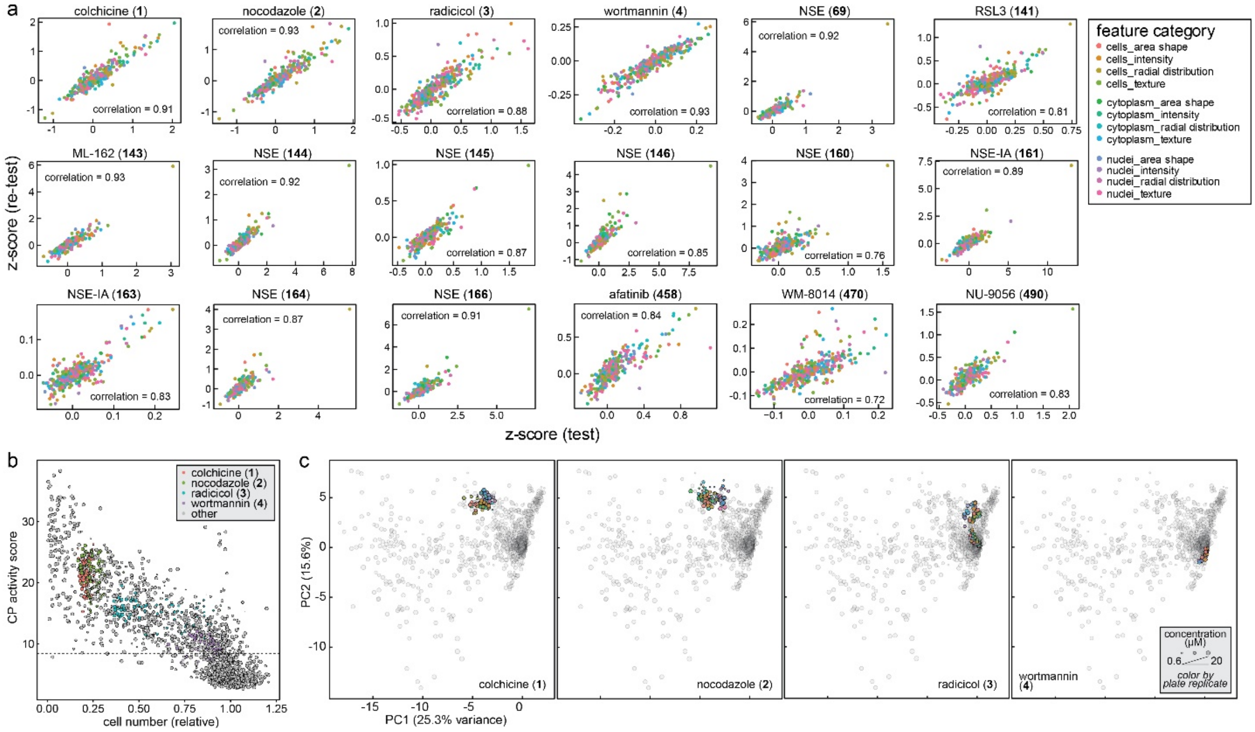

Supplementary Figure 3. Cell painting phenotypes show acceptable reproducibility. The performance of select cellular injury compounds and positive CP controls was evaluated to determine if cellular injury phenotypes were reproducible across independent experiments. (a) Select compounds show high correlations across independent CP experiments (test and re-test). The scatterplots depict the z-scores of each cellular feature, with points colored according to the feature category. (b, c) CP positive controls **1-4** show reproducible activities, cell numbers, and PCA clustering after 24 h treatment times across 13 compound plates (pooled from 2 independent experiments, 4 replicate plates per compound plate). CP, cell painting; PC, principal component. Source data are provided as a Source Data file.

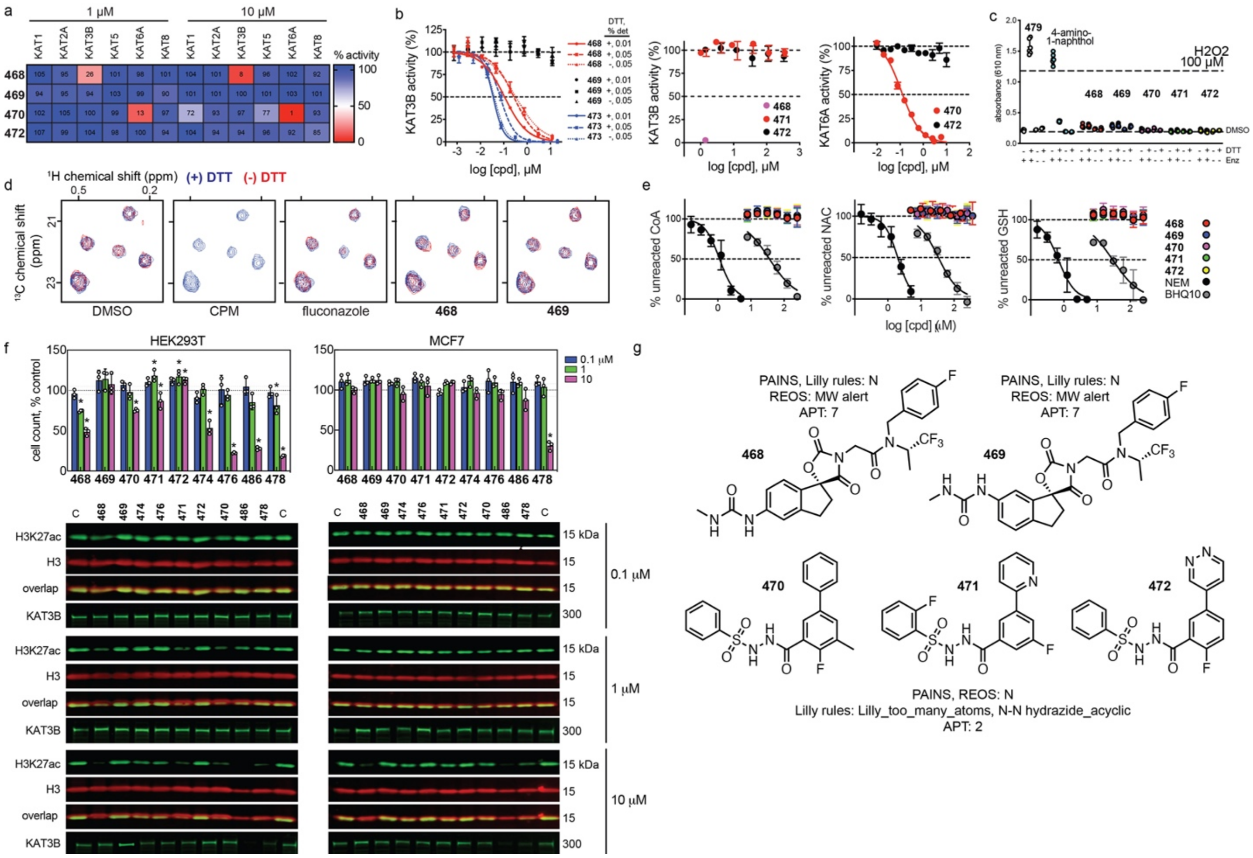

Supplementary Figure 4. Next-generation KAT inhibitors are high-quality chemical probes with minimal common assay interferences. The ngKATIs were profiled for common modes of assay interferences that have been associated with historical KATIs. (a) Compounds **468** and **470** are potent and selective cell-free inhibitors of KAT3B and KAT6A, respectively, in a KAT biochemical profiling panel. Data are mean of two intra-run technical replicates. (b) Compounds **468** and **470** are potent and selective cell-free inhibitors of KAT3B and KAT6A, respectively, in additional KAT biochemical assays performed in concentration-response format. Data are mean ± SD from three intra-run technical replicates. (c) Compounds **468-472** do not produce detectable levels of hydrogen peroxide. Data are mean ± SD from three (no enzyme) or six (enzyme) intra-run technical replicates performed on the same microplate. (d) Compounds **468** and **469** are ALARM NMR-negative, suggesting they are not grossly reactive with protein thiols. Signal intensities (*z*-axis, relative units) normalized to DMSO controls. (e) Compounds **468-472** do not react with the non-proteinaceous thiols coenzyme A (CoA), glutathione (GSH), or *N*-acetyl cysteine (NAC) by fluorometric thiol reactivity assay. Data are mean ± SD from six intra-run technical replicates performed on the same microplate. (f) Top: prototypical interference compounds, but not next-generation KAT inhibitors, disrupt HEK293T and MCF7 cell proliferation. Data are mean ± SD from three intraplate technical replicates. Bottom: the next-generation KAT inhibitor **468** and some prototypical interference compounds (**478**, **486**) decrease H3K27ac levels. (g) Compounds **468-472** exhibit do not exhibit red flags by cheminformatics analysis for substructure pan assay interference compounds (PAINS), rapid elimination of swill (REOS), Lilly alerts (FAF-Drugs4, accessed 11 July 2018) and Abbott Physicochemical Tiering (APT; Pipeline Pilot, BIOVIA, version 9.0.2.1). The clean interference profiles support grouping ngKATIs as distinct from hKATIs. C, control; DTT, dithiothreitol; Enz, enzyme; H3, histone H3; H3K27ac, histone H3 lysine 27 acetylation; KAT, lysine acetyltransferase. Source data are provided as a Source Data file.

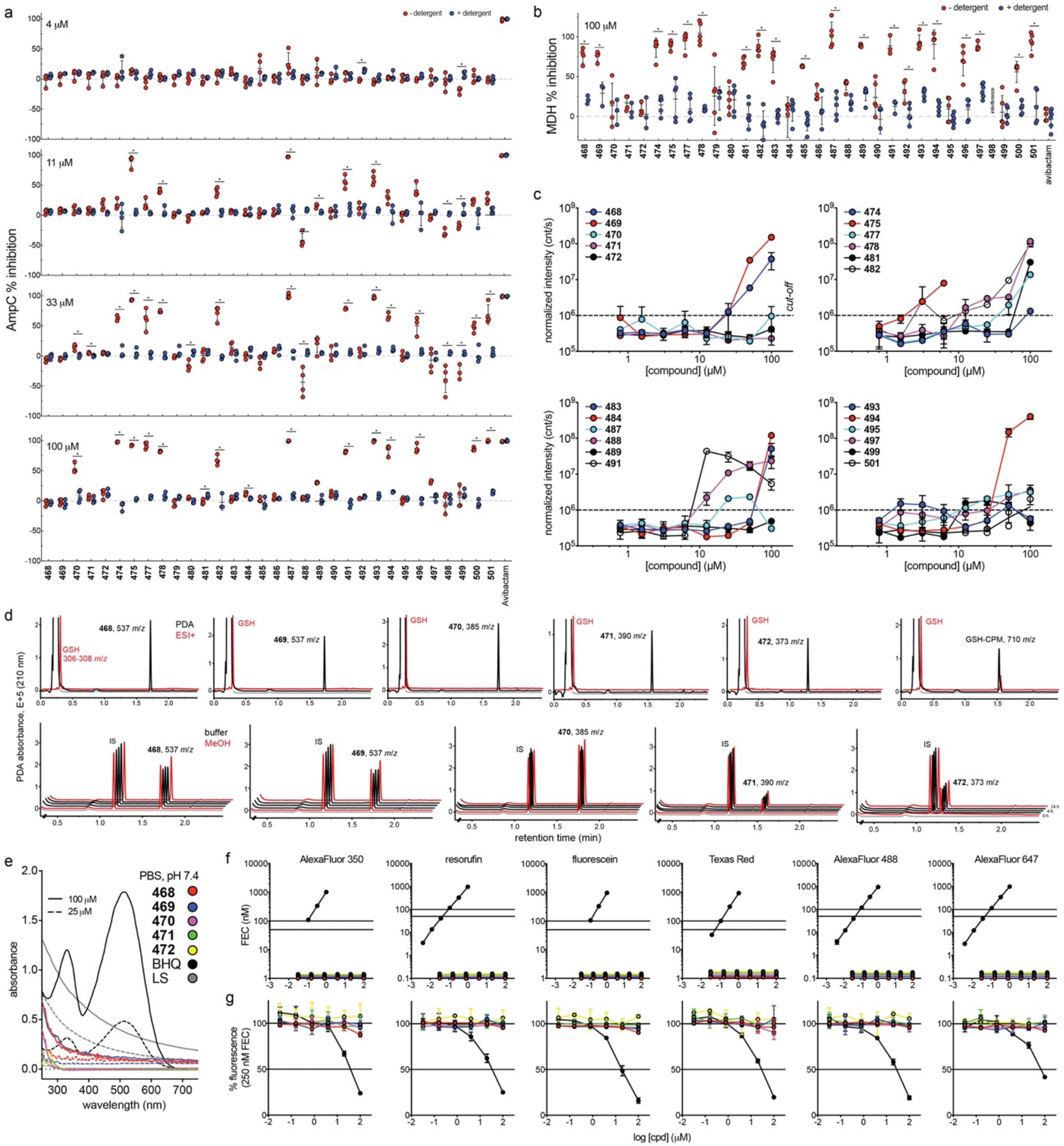

Supplementary Figure 5. Next-generation KAT inhibitors show minimal cell-free interferences by additional counter-screens. The ngKATIs were further profiled for common modes of assay interferences that have been associated with historical KATIs. (a, b) Many historical KAT inhibitors show detergent-sensitive inhibition of AmpC β-lactamase and malate dehydrogenase (MDH). Detergent-sensitive differences in inhibition are a hallmark of compounds that form colloidal aggregates. Data are individual replicates with bars denoting mean ± SD from four intra-run technical replicates. (c) Several KAT inhibitors form detectable colloidal aggregates by dynamic light scattering. Data are mean ± SD from three intra-run technical replicates performed on the same microplate. (d) Compounds **468-472** do not form grossly detectable adducts with

glutathione (GSH, top) and are grossly stable in assay-like conditions by ultra-performance liquid chromatography-mass spectrometry (bottom). (e) Compounds **468-472** do not significantly absorb visible light in phosphate-buffered saline (PBS). Positive control (PC), BHQ-10 carboxylic acid (λ_{max} = 516 nm); light scatterer (LS), creamer light scattering control (1 and 0.5 mg mL^{-1}). (f, g) Compounds **468-472** do not fluoresce or quench fluorescence in six common fluorophore channels. Dotted lines, upper/lower limit of quantification. PC auto-fluorescence, independent fluorophore sample; PC quenching, BHQ-10. Data are mean ± SD from three intra-run technical replicates performed on the same microplate**.** The clean interference profiles support grouping ngKATIs as distinct from hKATIs. Source data are provided as a Source Data file.

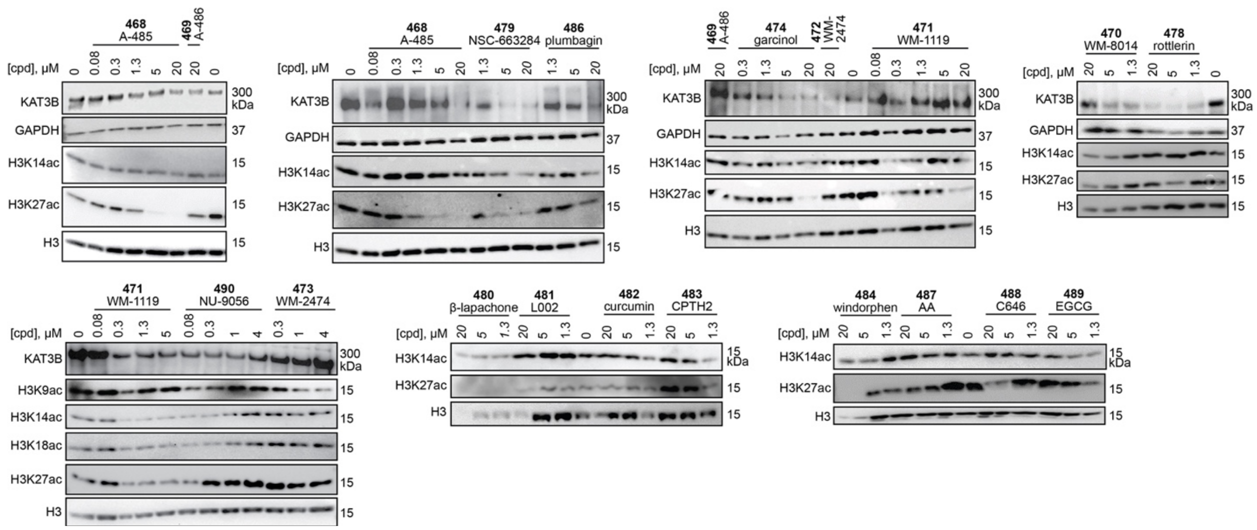

Supplementary Figure 6. Next-generation KAT inhibitors and prototypical nuisance compounds produce overlapping but distinct cellular histone acetylation phenotypes in cell painting-like conditions. Both ngKATIs and hKATIs were profiled by western blotting for their effects on cellular histone acetylation to assess the relationship between CP phenotype and KAT inhibition activity. (a) U-2 OS cells were treated with compounds for 24 h at the indicated concentrations under experimental conditions mimicking the CP assay. Compound **468** (but not inactive analog **469**) potently reduced cellular H3K27ac levels (approximate EC_{50} = 1 μM) and did not modulate H3K14ac or KAT3B levels. The KAT6-specific inhibitors **470** and **471** mildly decreased cellular H3K9ac, H3K14ac, and H3K27ac levels. Note that these compound predominately target KAT6A, which acetylates primarily H3K23. Consistent with other profiling trends (Supplementary Figure 3), several prototypical interference compounds and historical KAT inhibitors decreased H3K27ac levels, while also markedly reducing KAT3B levels in U-2 OS cells. *Italicized concentrations*, notable cell death. Histone H3 and GAPDH, H3Kac and KAT3B normalization controls, respectively. GADPH, glyceraldehyde 3-phosphate dehydrogenase; H3, histone H3; H3K9/14/23/27ac, histone H3 lysine 9/14/23/27 acetylation; KAT, lysine acetyltransferase. Data are blots from a single experiment. Source data are provided as a Source Data file.

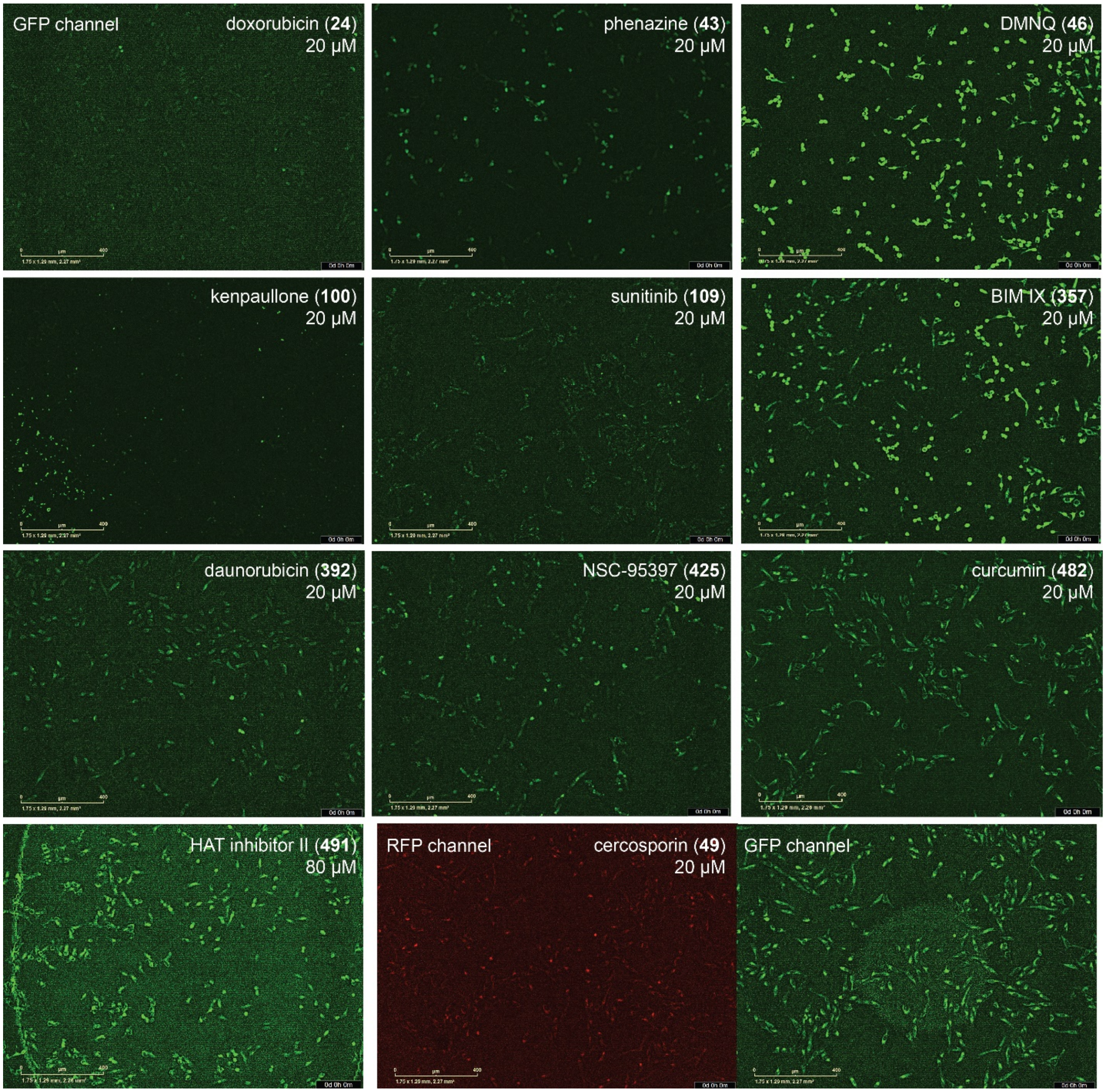

Supplementary Figure 7. Compound-dependent artifacts in live-cell imaging identified by reagent-free counter-screen. Representative examples of auto-fluorescent compound artifacts in live-cell imaging. U-2 OS cells were treated with compounds for 1 h in the absence of cellular health reagents, followed by imaging and processing otherwise identical to the primary live-cell cellular health assay. Note that most fluorescent compound artifacts interfered with the GFP (green) rather than the RFP (red) channel. Image scales: 400 μm. Data are representative images from three intra-run technical replicates each performed on separate microplates.

Supplementary Table 1. Sources of KAT inhibitors.

Compound	Name	Source	Notes
**468**	A-485	Abbvie	provided by SGC
**469**	A-486	Abbvie	provided by SGC
**470**	WM-8014	Monash University	synthesized in-house using published procedures[1,2].
**471**	WM-1119	Monash University	
**472**	WM-2474	Monash University	
**473**	Lys-CoA	National Center for Advancing Translational Sciences	
**474**	Garcinol	Cayman Chemical	cat # 10566
**475**	Isogarcinol	Cayman Chemical	cat # 21164
**476**	Dahlin-JMC2015-6a	UMN	
**477**	Embellin	Cayman Chemical	cat # 11838
**478**	Rottlerin	Cayman Chemical	cat # 12006
**479**	NSC-663284	Cayman Chemical	cat # 13303
**480**	Beta-lapachone	Santa Cruz Biotechnology	cat # sc-2000875
**481**	L002	Cayman Chemical	cat # 17778
**482**	Curcumin	Sigma-Aldrich	cat # 08511
**483**	CPTH2	Cayman Chemical	cat # 12086
**484**	Windorphen	Sigma-Aldrich	cat # SML0899
**485**	Windorphen-NC	Calbiochem	cat # 509164
**486**	Plumbagin	Cayman Chemical	cat # 14314
**487**	Anacardic acid	Cayman Chemical	cat # 13144
**488**	C646	Cayman Chemical	cat # 10549
**489**	EGCG	Cayman Chemical	cat # 70935
**490**	NU-9056	Tocris	cat # 4903
**491**	HAT Inhibitor II	Cayman Chemical	cat # 19835
**492**	Rtt109 Inhibitor	Enamine Ltd.	cat # Z14250979
**493**	MG149	Cayman Chemical	cat # 22135
**494**	EML425	EMD Millipore	cat # 534354
**495**	MB-3	Sigma-Aldrich	cat # M2449
**496**	SPV-106	Sigma-Aldrich	cat # SML0154
**497**	Gossypol	Cayman Chemical	cat # 14482
**498**	CTK7A	Sigma-Aldrich	cat # 382115
**499**	TH 1834	Axon Medchem	cat # 2339
**500**	Nordihydroguaiaretic acid	Sigma-Aldrich	cat # 74540
**501**	CAY10669	Cayman Chemical	cat # 10974

Supplementary Note 1. Summary of compound selection process. The cytotoxic and cellular injury compounds for cell painting profiling were selected by multiple criteria in accordance with previous recommendations.[3] These criteria include: (1) prototypical classes of cytotoxic compound classes (e.g., cytoskeletal poisons, nonspecific kinase inhibitors, HDAC inhibitors, genotoxins), (2) chemical availability and cost (deprioritizing long lead times and prioritizing low cost), and (3) vendor-supplied annotations of compound cytotoxicity and MoA (e.g., “cytotoxic”, “anti-tumor activity”, “nonselective”). The nonspecific electrophiles and historical KAT inhibitors were selected due to their well-documented nuisance behaviors from previous profiling efforts.[4,5]

Supplementary Note 2. Next-generation KAT inhibitors favorable by cheminformatics analyses. Literature analyses and scrutiny of the chemical structures of reported cell-active KAT3B and KAT6 tool compounds **468-472** raised minimal alerts. We note that substructure filters and calculated properties are only one of many tools for compound prioritization. None of these compounds were flagged as PAINS, though **468** and **469** were flagged by REOS filters for their higher MW. Lilly substructure filters flagged **470-472** for their hydrazide moieties (Supplementary Figure 3)[6-8]. Acylsulfonylhydrazides (acylsulfonohydrazides) have been viewed as problematic because of (1) the metabolic liability of the *N-N* bond, which can undergo facile cleavage or oxidation, (2) evidence that they form metal complexes, and (3) they can undergo facile nucleophilic attack under metal catalysis[9,10]. Compounds **470-472** showed favorable physicochemical properties by Abbott Physicochemical Tiering (APT), while **468** and **469** were tiered as less favorable because they are not Rule-of-Five compliant (Supplementary Figure 4)[11]. The latter is less concerning, as **468** has already been shown to be orally bioavailable[12]. Literature analysis showed that the oxazolidinedione core of **468** and **469**, although one might speculate might be mildly electrophilic with the potential to therefore be promiscuous, was not a frequent moiety reported in screening publications. Indeed, it is present in potent, selective, and convincing mineralcorticoid receptor antagonists, as well as the advanced kinase inhibitor, pegcantratinib[13,14]. Similarly, the sulfonoaroylhydrazide of **470-472**, while it could be speculated might bear a relatively electrophilic carbonyl group, did not appear significantly in screening literature and indeed has been reported by Pfizer to furnish potent inhibitors of human branched-chain amino transferase, with reasonable pharmacokinetic properties[15].

Supplementary Note 3. Compounds 468-472 show potent, on-target cell-free inhibition of their reported KAT targets (refer to Supplementary Figure 2b)**.** Compound **468** is a potent inhibitor of KAT3B activity (IC_{50} = 109 nM; complete response; non-cooperative Hill slope of 0.89, 95% CI 0.72 to 1.06) while **469** showed minimal activity. This is consistent with original reports (IC_{50} = 9.8 and 60 nM), which were obtained by TR-FRET and SPA assays, respectively[16]. Neither excess electrophilic scavenging agent dithiothreitol (DTT), nor nonionic detergent grossly altered the potency of **468**, suggesting it is not acting by thiol-reactivity or aggregation[17,18]. These potencies are several-fold weaker than Lys-CoA (**473**), a well-behaved yet cell-impermeable bi-substrate analog used as a positive control compound[2,19]. As expected for KAT6-specific inhibitors, neither **471** nor **472** showed appreciable activity versus KAT3B activity. Compound **470**, showed potent *in vitro* inhibition versus KAT6A in the SPA format (IC_{50} value of 114 $\pm$ 11 nM; complete response; non-cooperative Hill slope of 1.0). This is consistent with the original report (IC_{50} = 8 nM) obtained by an AlphaScreen assay.

Differences in cell-free IC_{50} values between the original report and this study are likely due to differences in assay design. For KAT3 inhibitors **468** and **469**, notable differences between the EMSA and previously published assay results include enzyme concentrations (0.2 versus 150 nM KAT3B, respectively), substrate concentrations (0.5 versus 1.0 μM acetyl-CoA, respectively), and protein construct (KAT3B BHC domain aa 1036-1822 versus KAT3B aa 1284-1673, respectively)[16]. For KAT6A/B inhibitors **470-472**, notable differences between the SPA and

previously published assay results include protein construct (KAT6A aa 497-780 versus aa 472-793, respectively), substrate concentrations (15 versus 5 μM acetyl-CoA, respectively), reaction buffer components, assay temperature, and reaction times[1].

Supplementary Note 4. Status of KAT3 in U-2 OS cells. There are 3 and 4 copies of KAT3A and KAT3B, respectively, and no KAT3 mutations present in U-2 OS cells according to the Cancer Cell Line Encyclopedia[20]. The *KAT3B* gene is not under-expressed in osteosarcoma cells according to the Broad Institute DepMap[21]. The protein KAT3B is also expressed in U-2 OS cells according to the MaxQB proteomics database[22].

Supplementary Note 5. Plate layout of typical cell painting experiment. Each 384-well CP plate contains 116 solvent/negative control wells (DMSO), four positive control compounds (**1**-**4**; colchicine, nocodazole, radicicol, wortmannin), and 168 wells available for test compounds. Most compounds were profiled at six compound concentrations, diluted row-wise (e.g., C3 to C8, 20 to 0.625 μM final concentrations, respectively).

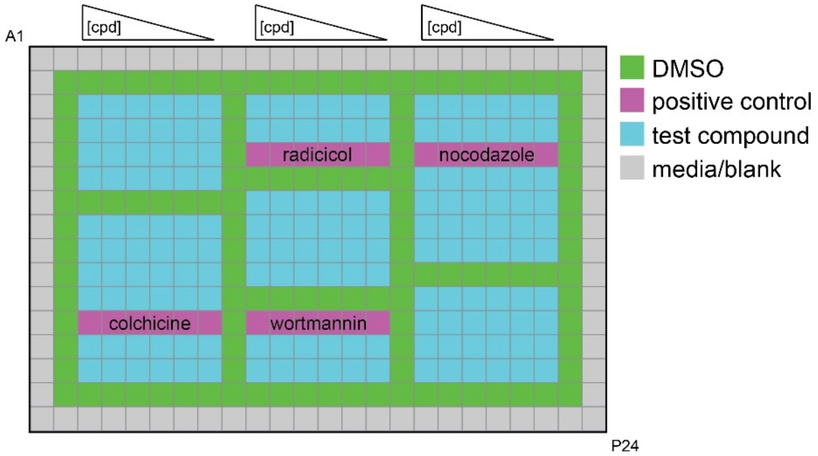

SUPPLEMENTARY REFERENCES

1. Baell, J. *et al.* Inhibitors of histone acetyltransferases KAT6A/B induce senescence and arrest tumour growth. *Nature* **560,** 253–257 (2018).
2. Lau, O.D. *et al.* HATs off: selective synthetic inhibitors of the histone acetyltransferases p300 and PCAF. *Mol. Cell.* **5,** 589–595 (2000).
3. Dahlin, J.L. *et al.* Nuisance compounds in cellular assays. *Cell Chem Biol* **28,** 356-370 (2021).
4. Dahlin, J.L. *et al.* PAINS in the assay: chemical mechanisms of assay interference and promiscuous enzymatic inhibition observed during a sulfhydryl-scavenging HTS. *J. Med. Chem.* **58,** 2091–2113 (2015).
5. Dahlin, J.L. *et al.* Assay interference and off-target liabilities of reported histone acetyltransferase inhibitors. *Nat. Commun.* **8,** 1527 (2017).
6. Walters, W.P. & Namchuk, M. Designing screens: how to make your hits a hit. *Nat. Rev. Drug Discov.* **2,** 259–266 (2003).
7. Baell, J.B. & Holloway, G.A. New substructure filters for removal of pan assay interference compounds (PAINS) from screening libraries and for their exclusion in bioassays. *J. Med. Chem.* **53,** 2719–2740 (2010).
8. Bruns, R.F. & Watson, I.A. Rules for identifying potentially reactive or promiscuous compounds. *J. Med. Chem.* **55,** 9763–9772 (2012).
9. Attanasi, O., Battistoni, P. & Fava, G. Effect of metal ions in organic synthesis: Part XII: a new synthesis of *N*-(α-chlorobenzylidene)-*N'*-phenylhydrazine by reaction of phenylazostilbene and copper(II) chloride. *Org. Prep. Proced. Int.* **15,** 1-5 (1983).
10. Goh, H., Nam, T.K., Singh, A., Singh, N. & Jang, D.O. Dipodal colorimetric sensor for Ag+ and its resultant complex for iodide sensing using a cation displacement approach in water. *Tetrahedron Lett.* **58,** 1040–1045 (2017).
11. Cox, P.B., Gregg, R.J. & Vasudevan, A. Abbott Physicochemical Tiering (APT) - a unified approach to HTS triage. *Bioorg. Med. Chem.* **20,** 4564–4573 (2012).
12. Michaelides, M. *et al.* Discovery of spiro oxazolidinediones as selective, orally bioavailable inhibitors of p300/CBP histone acetyltransferases. *ACS Med. Chem. Lett.* **9,** 28–33 (2017).
13. Yang, C. *et al.* Discovery of novel oxazolidinedione derivatives as potent and selective mineralocorticoid receptor antagonists. *Bioorg. Med. Chem. Lett.* **23,** 4388–4392 (2013).
14. Cranston, A. *et al.* Tropomyosin Receptor Antagonism in Cylindromatosis (TRAC), an early phase trial of a topical tropomyosin kinase inhibitor as a treatment for inherited CYLD defective skin tumours: study protocol for a randomised controlled trial. *Trials* **18,** 111 (2017).
15. Hu, L. *et al.* The design and synthesis of human branched-chain amino acid aminotransferase inhibitors for treatment of neurodegenerative diseases. *Bioorg. Med. Chem. Lett.* **16,** 2337–2340 (2006).
16. Lasko, L. *et al.* Discovery of a selective catalytic p300/CBP inhibitor that targets lineage-specific tumours. *Nature* **550,** 128–132 (2017).
17. Huth, J.R. *et al.* ALARM NMR: a rapid and robust experimental method to detect reactive false positives in biochemical screens. *J. Am. Chem. Soc.* **127,** 217–224 (2005).
18. Dahlin, J.L. *et al.* Post-HTS case report and structural alert: promiscuous 4-aroyl-1,5-disubstituted-3-hydroxy-2*H*-pyrrol-2-one actives verified by ALARM NMR. *Bioorg. Med. Chem. Lett.* **25,** 4740–4752 (2015).
19. Liu, X. *et al.* The structural basis of protein acetylation by the p300/CBP transcriptional coactivator. *Nature* **451,** 846–850 (2008).
20. Barretina, J. *et al.* The Cancer Cell Line Encyclopedia enables predictive modelling of anticancer drug sensitivity. *Nature* **483,** 603-607 (2012).
21. Tsherniak, A. *et al.* Defining a Cancer Dependency Map. *Cell* **170,** 564-576.e16 (2017).

22. Schaab, C., Geiger, T., Stoehr, G., Cox, J. & Mann, M. Analysis of high accuracy, quantitative proteomics data in the MaxQB database. *Mol Cell Proteomics* **11,** M111.014068 (2012).
