# Supplementary material for: Reference compounds for characterizing cellular injury in high-content cellular morphology assays
Source: Nat Commun. 2023 Mar 13;14:1364. doi: 10.1038/s41467-023-36829-x (PMC10011410; doi:10.1038/s41467-023-36829-x)
Supplement: Supplementary file 3 — Reporting Summary [file 41467_2023_36829_MOESM3_ESM.pdf]

nature portfolio

Corresponding author(s): Jayme L Dahlin
Bridget K Wagner

Last updated by author(s): Feb 17, 2023

Reporting Summary

Nature Portfolio wishes to improve the reproducibility of the work that we publish. This form provides structure for consistency and transparency in reporting. For further information on Nature Portfolio policies, see our Editorial Policies and the Editorial Policy Checklist.

Statistics

For all statistical analyses, confirm that the following items are present in the figure legend, table legend, main text, or Methods section.

n/a	Confirmed	
☐	☒	The exact sample size (n) for each experimental group/condition, given as a discrete number and unit of measurement
☐	☒	A statement on whether measurements were taken from distinct samples or whether the same sample was measured repeatedly
☐	☒	The statistical test(s) used AND whether they are one- or two-sided<br>*Only common tests should be described solely by name; describe more complex techniques in the Methods section.*
☐	☒	A description of all covariates tested
☐	☒	A description of any assumptions or corrections, such as tests of normality and adjustment for multiple comparisons
☐	☒	A full description of the statistical parameters including central tendency (e.g. means) or other basic estimates (e.g. regression coefficient) AND variation (e.g. standard deviation) or associated estimates of uncertainty (e.g. confidence intervals)
☐	☒	For null hypothesis testing, the test statistic (e.g. F, t, r) with confidence intervals, effect sizes, degrees of freedom and P value noted<br>*Give P values as exact values whenever suitable.*
☒	☐	For Bayesian analysis, information on the choice of priors and Markov chain Monte Carlo settings
☒	☐	For hierarchical and complex designs, identification of the appropriate level for tests and full reporting of outcomes
☐	☒	Estimates of effect sizes (e.g. Cohen's d, Pearson's r), indicating how they were calculated

Our web collection on statistics for biologists contains articles on many of the points above.

Software and code

Policy information about availability of computer code

Data collection	PerkinElmer Harmony (version 4.9); Wyatt DYNAMICS (version 1.7)<br><br>No custom algorithms, code, or software was used for data collection.
Data analysis	CellProfiler (version 2.1.1); R (version 3.6.1); GraphPad Prism (version 8.4.2); Incucyte Analysis software (version 2018B); Wyatt DYNAMICS (version 1.7); Bruker TopSpin (version 4.0.7); MestReNova (version 14.1.0-24037)<br><br>No custom algorithms, code, or software was used for data analysis

For manuscripts utilizing custom algorithms or software that are central to the research but not yet described in published literature, software must be made available to editors and reviewers. We strongly encourage code deposition in a community repository (e.g. GitHub). See the Nature Portfolio guidelines for submitting code & software for further information.

Data

Policy information about availability of data

All manuscripts must include a data availability statement. This statement should provide the following information, where applicable:

- Accession codes, unique identifiers, or web links for publicly available datasets
- A description of any restrictions on data availability
- For clinical datasets or third party data, please ensure that the statement adheres to our policy

The multi-terabyte collection of CP images, metadata, and associated CellProfiler object-level files generated in this study have been deposited in the Image Data Resource database under accession code idr0133 [https://idr.openmicroscopy.org]. The processed CP extracted feature data, the processed live-cell imaging data, the processed intracellular glutathione data, the raw ALARM NMR spectra, and the raw UPLC-MS data for KAT inhibitors have been deposited in the Figshare database under accession code 20293992 [https://doi.org/10.6084/m9.figshare.20293992.v1]. Source data are provided as a Source Data file. Key descriptors (categories, SMILES, purity, annotations) for study compounds and the composition of the proposed cellular injury informer set are provided in Supplementary Data 1.

Human research participants

Policy information about studies involving human research participants and Sex and Gender in Research.

Reporting on sex and gender	Not applicable. No human research was performed in this study.
Population characteristics	Not applicable. No human research was performed in this study.
Recruitment	Not applicable. No human research was performed in this study.
Ethics oversight	Not applicable. No human research was performed in this study.

Note that full information on the approval of the study protocol must also be provided in the manuscript.

Field-specific reporting

Please select the one below that is the best fit for your research. If you are not sure, read the appropriate sections before making your selection.

[x] Life sciences [ ] Behavioural & social sciences [ ] Ecological, evolutionary & environmental sciences

For a reference copy of the document with all sections, see nature.com/documents/nr-reporting-summary-flat.pdf

Life sciences study design

All studies must disclose on these points even when the disclosure is negative.

Sample size	For cell painting, the sample size of four technical replicates were chosen based on previous recommendations (PMID 27560178). Compounds were also tested in concentration-response format (usually six concentrations). For other experiments, the number of technical replicates (usually three) are sufficient to determine significant differences between compounds within a high-throughput experiment.
Data exclusions	No data were excluded from the analyses.
Replication	Most compounds in this study were profiled by high-throughput methods that rely on technical replicates. Compounds were also tested in concentration-response format. Select compounds were tested in multiple independent cell painting experiments with acceptable reproducibility (Supplementary Figure 3).
Randomization	Not applicable. No animal studies were performed in this work.
Blinding	Not applicable. All biological samples were analyzed equally.

Reporting for specific materials, systems and methods

We require information from authors about some types of materials, experimental systems and methods used in many studies. Here, indicate whether each material, system or method listed is relevant to your study. If you are not sure if a list item applies to your research, read the appropriate section before selecting a response.

Materials & experimental systems

n/a	Involved in the study
☐	☒ Antibodies
☐	☒ Eukaryotic cell lines
☒	☐ Palaeontology and archaeology
☒	☐ Animals and other organisms
☒	☐ Clinical data
☒	☐ Dual use research of concern

Methods

n/a	Involved in the study
☒	☐ ChIP-seq
☒	☐ Flow cytometry
☒	☐ MRI-based neuroimaging

Antibodies

Antibodies used

For MCF7 and HEK293T experiments, the following antibodies were used:
H3K27ac (Cell Signaling Technologies, cat # 8173, dilution 1:1,000)
H3 (Abcam, cat # 10799, dilution 1:1,000)
KAT3B (P300, Bethyl, cat # A300-358A, dilution 1:2,000)
Goat anti-rabbit IgG (IRDye 800-conjugated, LI-COR, cat # 926-32211, dilution 1:5,000)
Donkey anti-mouse IgG (IRDye 680-conjugated, LI-COR, cat # 926-68072, dilution 1:5,000)

For U-2 OS experiments, the following antibodies were used:
KAT3B (P300, Bethyl, cat # A300-358A, dilution 1:10,000)
GAPDH (CST, cat # 2118S, dilution 1:2,000)
H3 (Abcam, cat # AB1791, dilution 1:5,000)
H3K14ac (Millipore, cat # 07-353, dilution 1:2,000)
H3K27ac (Cell Signaling Technologies, cat # 8173, dilution 1:2,000)
Anti-rabbit HRP-conjugated secondary antibody (Cell Signaling Technologies, cat # 7074S, dilution 1:5,000)

Validation

Performed western blots per manufacturer instructions. Manufacturer-provided validation materials can be found at:

H3K27ac, Cell Signaling Technologies, https://media.cellsignal.com/pdf/8173.pdf
H3, Abcam, https://www.abcam.com/histone-h3-antibody-mabcam-10799-chip-grade-ab10799.pdf
KAT3B, Bethyl, https://www.bethyl.com/product/A300-358A/p300+Antibody
Goat anti-rabbit IgG, LiCor, https://www.licor.com/documents/rfm2hw40wf33p06f3ndjrcorwi5usbft
Donkey anti-mouse IgG, LiCor, https://www.licor.com/documents/1rur5wg8vsznx2ll28hgimwzjsgrlbsf

GAPDH, Cell Signaling Technologies, https://media.cellsignal.com/pdf/2118.pdf
H3, Abcam, https://www.abcam.com/histone-h3-antibody-nuclear-loading-control-and-chip-grade-ab1791.pdf
H3K14ac, Millipore, https://www.emdmillipore.com/US/en/product/Anti-acetyl-Histone-H3-Lys14-Antibody,MM_NF-07-353#anchor_COA
H3K27ac, Cell Signaling Technologies, https://media.cellsignal.com/pdf/8173.pdf
Anti-rabbit HRP-conjugated secondary antibody, Cell Signaling Technologies, https://media.cellsignal.com/pdf/7074.pdf

Eukaryotic cell lines

Policy information about cell lines and Sex and Gender in Research

Cell line source(s)

HEK293T (gifr from Dr. Sam Benchimol, York University; ATCC, cat # CRL-3216)
MCF7 (ATCC, cat # HTB-22)
U-2 OS (ATCC, cat # HTB-96)

Authentication

All cell lines were obtained from trusted sources and authenticated by STR profiling (ATCC Cell Line Authentication Service or provided by vendor) upon receipt.

Mycoplasma contamination

All cell lines (HEK293T, MCF7, U-2 OS) were tested negative for Mycoplasma contamination (MycoAlert Mycoplasma Detection Kit, Lonza, cat # LT07-701). Testing was performed monthly.

Commonly misidentified lines (See ICLAC register)

None of the cell lines (HEK293T, MCF7, U-2 OS) are commonly misidentified lines per ICLAC registry.
