# Supplementary material for: Reference compounds for characterizing cellular injury in high-content cellular morphology assays
Source: Nat Commun. 2023 Mar 13;14:1364. doi: 10.1038/s41467-023-36829-x (PMC10011410; doi:10.1038/s41467-023-36829-x)
Supplement: Supplementary file 4 — Description of Additional Supplementary Files [file 41467_2023_36829_MOESM4_ESM.pdf]

Title: Supplementary Data 1
Description: Key compound descriptors (categories, SMILES, purity, annotations) for study compounds and proposed cellular injury informer set (XLSX).
